# Factors associated with the isolation of Nontuberculous mycobacteria (NTM) from a large municipal water system in Brisbane, Australia

**DOI:** 10.1186/1471-2180-13-89

**Published:** 2013-04-22

**Authors:** Rachel M Thomson, Robyn Carter, Carla Tolson, Chris Coulter, Flavia Huygens, Megan Hargreaves

**Affiliations:** 1 Gallipoli Medical Research Centre, Greenslopes Private Hospital, Brisbane, QLD, Australia; 2 QLD Mycobacterial Reference Laboratory, Pathology Queensland, RBWH Campus, Herston Rd, Herston, QLD 4006, Australia; 3 Queensland University of Technology, Institute of Health and Biomedical Innovation, Kelvin Grove Campus, Brisbane, QLD 4059, Australia; 4 Queensland University of Technology, Faculty of Science and Technology, George Street, Brisbane, QLD 4001, Australia

## Abstract

**Background:**

Nontuberculous mycobacteria (NTM) are normal inhabitants of a variety of environmental reservoirs including natural and municipal water. The aim of this study was to document the variety of species of NTM in potable water in Brisbane, QLD, with a specific interest in the main pathogens responsible for disease in this region and to explore factors associated with the isolation of NTM. One-litre water samples were collected from 189 routine collection sites in summer and 195 sites in winter. Samples were split, with half decontaminated with CPC 0.005%, then concentrated by filtration and cultured on 7H11 plates in MGIT tubes (winter only).

**Results:**

Mycobacteria were grown from 40.21% sites in Summer (76/189) and 82.05% sites in winter (160/195). The winter samples yielded the greatest number and variety of mycobacteria as there was a high degree of subculture overgrowth and contamination in summer. Of those samples that did yield mycobacteria in summer, the variety of species differed from those isolated in winter. The inclusion of liquid media increased the yield for some species of NTM. Species that have been documented to cause disease in humans residing in Brisbane that were also found in water include *M. gordonae, M. kansasii, M. abscessus, M. chelonae, M. fortuitum complex, M. intracellulare, M. avium* complex, *M. flavescens, M. interjectum, M. lentiflavum, M. mucogenicum, M. simiae, M. szulgai, M. terrae. M. kansasii* was frequently isolated, but *M. avium* and *M. intracellulare* (the main pathogens responsible for disease is QLD) were isolated infrequently. Distance of sampling site from treatment plant in summer was associated with isolation of NTM. Pathogenic NTM (defined as those known to cause disease in QLD) were more likely to be identified from sites with narrower diameter pipes, predominantly distribution sample points, and from sites with asbestos cement or modified PVC pipes.

**Conclusions:**

NTM responsible for human disease can be found in large urban water distribution systems in Australia. Based on our findings, additional point chlorination, maintenance of more constant pressure gradients in the system, and the utilisation of particular pipe materials should be considered.

## Background

The environmental or nontuberculous mycobacteria (NTM) are a group of human and animal pathogens that have significant impacts on the morbidity and mortality of humans and important economic impacts on agriculture [1]. They are normal inhabitants of a variety of environmental reservoirs including natural and municipal water, soil, aerosols, and protozoans. The incidence of pulmonary disease due to environmental mycobacteria is increasing in many parts of the world including Queensland [2]. Clinically significant cases represent approximately one-third of all NTM pulmonary patient-isolates processed by laboratories in the state. Postulated reasons for this increase include increased awareness of mycobacteria as pulmonary pathogens, improvements in methods of detection and culture, and an ageing population (as this is often a disease of the elderly).

It has been shown that mycobacteria are resident in drinking water distribution systems [3-8]. They have also been found in hospital water distribution systems [9-11] and domestic tap water [12-16]. In 2007, Brisbane Water managed the supply of potable water to the population of Brisbane. Details of the system supplying water to approximately 1 million residents are tabled in Additional file 1: Table S1. Water is treated by chloramination at the treatment plants and by point chlorination at points of entry into the system. There are 45 Reservoirs within the network.

There have been no published studies examining the presence of mycobacteria in water distribution systems in Australia, and routine sampling/monitoring is not mandated as part of public health practice.

The aim of this study was to determine if mycobacteria that currently cause disease in Queensland residents and those that are found as contaminants in human samples, are present in Brisbane drinking water and to explore system factors that may be associated with the presence or absence of these NTM.

## Methods

Water authorities routinely sample approximately 220 sites across Brisbane as part of water quality maintenance. These sites are a mixture of Trunk Main (TM) samples, Reservoir (R) samples, and Distribution point (D) samples. For this study an extra litre (sample) was collected from each site to allow filtration and culture for mycobacteria. Samples were collected in 1Litre sterile bottles and transported at 4**°**C to the QLD Mycobacterial Reference Laboratory.

Samples were collected over a 3-week period in both Winter (July-August 2007) and Summer (December 2007-January 2008). Each sample was halved, with 500 ml treated with 0.005% Cetylpyridinium chloride (CPC) for 30 minutes. Filtration was performed through 0.45 μm cellulose nitrate filters (Sartorius AG 37070 Goettingen, Germany). Filters were then rinsed with 2ml sterile distilled water (SDW) and macerated and then 0.1ml aliquots were then transferred in triplicate to Middlebrook 7H11 plates, which were sealed in gas permeable plastic bags for incubation at 32°C. For the winter samples 0.5 ml aliquots were also transferred to two Mycobacterial Growth Indicator Tubes (MGITs), one containing PANTA (polymixin, azlocillin, nalidixic acid, trimethoprim, amphotericin B) and incubated using the Bactec 960 system (Becton Dickinson, North Ryde, NSW). As a result, each sample collected in winter resulted in 10 processed cultures and each sample in summer resulted in six processed cultures.

Plates were inspected weekly and a representative selection of each morphological type of Ziehl Nielsen (ZN) positive colonies from each site sample were subcultured onto 7H11 plates. Multiplex PCR [17] was performed followed by 16S-rRNA sequencing of mycobacterial isolates and compared using RIDOM and GenBank database [18,19]. Sequence homology of ≥97% was accepted. Those identified as *M. abscessus/M. chelonae* underwent *hsp*65 and *rpo*B sequencing for more definitive identification. Because of the widely varying growth rates of different NTM species, and the presence of multiple different colony types and species in samples from each site, determination of concentrations of individual NTM species in CFU/ml was not determined. The number of different species/strains from each site was determined and expressed per site (1L sample) for each season.

Information regarding the different sampling sites was obtained and included mains age, pipe material, distance from nearest reservoir, and elevation above sea level. Distances between main treatment plants and sampling sites were calculated from latitude and longitude values provided for each site.

Statistics: Statistical analysis was performed using IBM SPSS v 20.

Sampling site variables were analysed against individual site culture results using a one-way ANOVA with post hoc Bonferroni correction. Culture method variables were analysed against results of individual replicates. Where variables were not normally distributed, the non parametric Kruskal Wallis test was used. Elevation above sea level was transformed (square root) and analysed using one way ANOVA. Categorical variables were analysed using Chi Square rxc tables. Values are represented as mean +/- SD or median (where non normal distribution); significance level p = 0.05.

## Results

### Sampling site analysis

Of a total of 217 sites, 1L-samples from 189 sites in summer and 195 sites in winter were received. Because of the drought conditions experienced in QLD at the time of the study and subsequent water restrictions, 17 of the sampling sites were dry during summer and not able to be sampled. An additional 11 sites were therefore recruited that had not been part of the sampling routine during the preceding winter. Overall mycobacteria were identified in 61.5% samples. Mycobacteria were grown from 40.2% sites in summer (76/189) and 82.1% sites in winter (160/195). The lower yield in summer was due to higher rates of contamination, including that of subculture plates. Of the colonies subcultured and sequenced, 236 colonies were subsequently identified as NTM. Winter yields were greater (Mean 2.59 ± 1.62 colonies per site sample; range 1–10) compared with summer (1.70 ± 0.84; 1–4).

For those sites that were supplied water from Mt Crosby (152 sites in summer, 158 sites in winter), the distance of the sampling site from the treatment plant was associated with culture result particularly in summer; the mean distance from plant to site was 81.75 ± 6.99 km for negative sites, 82.50 ± 6.17 km for contaminated/overgrown sites and 85.40 ± 6.46 km for positive sites (p = 0.015). In winter the distances were similar (negative 84.95 ± 6.77km; contaminated/overgrown 82.49 ± 6.77 km; positive 83.34 ± 6.65 km; p = 0.581).

For those 17 sites receiving water from the Pine treatment plant or from both treatment plants (19 summer, 17 winter), the distance of sampling site from the treatment plant didn’t correlate with culture result.

### Type of sample

Samples came from distribution points (D), reservoirs (R) or trunk mains (TM). By their nature, the samples differed significantly according to differences in pipe diameter, and pipe material. The characteristics of the different type of samples are shown in Additional file 2: Figure S1 and Table S2. The majority of Trunk Main samples (also larger diameter) were of Mild Steel Cement Lined (88%), the remainder were Cast iron spun lined (6.6%), cast iron cement lined (3.3%) or Mild steel unlined black piping (2.1%). Reservoir samples similarly came mostly from Mild Steel Cement lined pipes (75.5%), with the remainder from Cast Iron spun lined (13%), Cast Iron Cement Lined (4.3%), Asbestos Cement (2.2%) or Ductile Iron Cement Lined (2.2%) In contrast the majority of distribution samples came from Asbestos cement or Cast Iron Spun lined pipes.

There was no statistically significant difference between culture results overall and sample type (ie Distribution/Reservoir/Trunk Main), mains age, pipe material or distance of sampling site from nearest reservoir (Table 1).This held true when winter and summer samples were analysed separately, though there was a trend towards more positive sites that were distribution samples (p = 0.074) with narrower diameter pipes in winter (p = 0.114). Whilst there were differences in the culture results from different pipe materials the numbers in some categories were too small to be statistically meaningful.

**Table 1:** Summary of NTM positive and negative sampling site variables

	**NTM Negative**	**NTM Positive**	**Significance (p value)**
Sampling Site Factor (Mean ± SD)			
**Site elevation** (meters above sea level)*	44.75 ± 40.12	43.78 ± 39.99	0.977
S	44.94 ± 41.92	44.88 ± 38.86	0.680
W	43.51 ± 26.54	43.26 ± 40.63	0.751
**Pipe Diameter** (cm)	438.01 ± 459.91	435.21 ± 461.92	0.954
S	403.23 ± 417.56	489.15 ± 513.25	0.211
W	553.94 ± 571.58	409.59 ± 434.81	0.103
**Mains Age** (years)	46.56 ± 19.53	48.94 ± 19.15	0.246
S	46.15 ± 19.83	50.97 ± 17.74	0.091
W	47.91 ± 18.71	47.97 ± 19.77	0.987
**Pipe material**			
Asbestos cement	28 (30.8%	63 (69.2)	0.166
Cement lined†	77 (41.8)	107 (58.2)	
PVC	6 (42.9)	8 (57.1)	
Cast iron spun lined	30 (35.7)	54 (64.3)	
Other‡	7 (63.3)	4 (36.4)	
**Sample type N (%)**			
Distribution	86 (37.1)	146 (62.9)	0.668
Reservoir	36 (39.1)	56 (60.9)	
Trunk Main	26 (43.3)	34 (56.7)	
**Surface water source N (%)**			
Mt Crosby	120 (38.6)	191 (61.4)	0.995
Pine	14 (37.8)	23 (62.2)	
Mixed	14 (38.9)	22 (61.1)	

*Elevation non normally distributed, square root transformation to analyse.

†Cast iron, ductile iron or mild steel cement lined.

‡Steel unlined/ polyethylene/unknown.

Trunk Main samples grew *M. kansasii, M. gordonae, M. mucogenicum, M. abscessus, M. chelonae, M. lentiflavum, M. simiae, M. szulgai, M. fortuitum* complex, and hence these species are also potentially present in more distal sites.

Some species relevant to humans, namely *M. intracellulare, and M. flavescens* were grown from reservoir samples though may not have been detected more distally in distribution point samples because of the limitations of culture techniques (overgrowth, contamination etc.). (Additional file 3: Species of NTM isolated from different sample types)

All variables were examined between different species of NTM. Pathogenic NTM (defined as those that had been found in human samples in QLD and known to cause disease) were more likely to be identified from sites with narrower diameter pipes, predominantly distribution sample points, and from sites with asbestos cement or modified PVC pipes. No other variables were found to be significant (Table 2).

**Table 2:** Presence of pathogenic NTM against different variables

**Variable**	**Pathogenic NTM**	**Non pathogenic NTM**	**P value**
**Sample type**			**0.001**
Distribution	203	129	
Reservoir	56	75	
Treatment Plant	33	41	
**Surface water source**			0.695
Crosby	231	195	
Mixed	25	25	
Pine	36	26	
Distance to nearest reservoir (km) Mean (±SD)	4.46 (5.01)	4.85 (6.18)	0.423
Age of water mains (yrs) Mean (±SD)	49.45 (19.50)	49.03 (19.11)	0.646
Pipe diameter (mm) Mean (±SD)	360.82 (414.90)	509.74 (503.47)	**<0.0001**
Site elevation	43.26 (45.50)	44.97 (37.17)	0.638
**Pipe material**			
Asbestos cement	91 (62.3)	55 (37.7)	**0.046**
Cast iron cement lined	26 (56.5)	20 (43.5)	
Cast iron spun lined	68 (59.1)	47 (40.9)	
Ductile Iron cement lined	14 (50)	14 (50)	
Mild steel cement lined	75 (44.4)	95 (55.9)	
Mild steel unlined black	3 (42.9)	4 (57.1)	
Modified PVC	5 (88.3)	1 (16.7)	
Polyethylene	0	1 (100)	
Unplasticized PVC	8 (61.5)	5 (38.5)	

### Location

The reservoir zones that cluster around the Central Brisbane District (CBD) appeared to contribute more positive sites than those in more peripheral zones (ie had more positive sites relative to the proportion of sites sampled) however this did not meet statistical significance. Of the sites within an approximate 5-kilometre radius of the CBD, 64.8% grew NTM, compared to 59.9% of sites outside this area (p = 0.431 Fisher’s exact test).

### Methodological factors associated with positive culture results

To assess the effect of decontamination and the relative contribution of the different media to positive results and species variety, the individual results of each culture taken per site was analysed. The results were analysed for summer and winter separately as contamination issues in summer would have confounded the result.

In winter, there were 10 cultures per site, and in summer 6 cultures per site. Hence, there were 1176 plates and 784 MGITs processed in winter (with PANTA added to half of these) and 1140 7H11 plates were processed in summer. For funding reasons, MGITs were not used in summer. Overall 65.3% of cultures were positive for mycobacterial growth, though there were statistically significant differences between summer and winter (p < 0.0001).

### Winter

Of 1960 cultures processed during winter, 528 (26.9%) failed to grow any colonies and 188 (9.6%) were overgrown to the extent that mycobacteria could not be detected, if they were present; 847 (43.2%) of cultures had positive growth and 397 (20.3%) were positive but with contaminants (presumed fungal on the basis of plate morphology, but not formally identified). The winter cultures yielded the greatest number and variety of mycobacteria (Table 3). This held true even if MGIT samples were excluded, though there were some specific contributions of the liquid media discussed below.

**Table 3:** Species of NTM identified in water samples collected in winter and summer

	**Summer**	**Winter**
*M. abscessus*	2	11
*M. abscessus/chelonae*		1
*M. angelicum/szulgai*		1
*M. arupense*	4	5
*M. austroafricanum*	1	
*M. bolletii/M. massiliense*		1
*M. chelonae*		2
*M. cookii*	1	1
*M. cosmeticum*	1	1
*M. diernhoferi*	1	1
*M. farcinogenes*	1	2
*M. flavescens*	2	1
*M. fluoranthenivorans*	11	4
*M. fortuitum complex*	13	14
*M. gadium*	1	4
*M. gilvum*	1	
*M. gordonae*	24	120
*M. interjectum*	1	7
*M. intracellulare*		2
*M. kansasii*	5	133
*M. lentiflavum*		19
*M. mageritense*	1	4
*M. moriokaense*		1
*M. mucogenicum*	31	42
*M. poriforae*	18	6
*M. rhodesiae*		1
*M. sengalense*	2	
*M. simiae*		2
*M. species NFI*	5	4
*M. terrae*	2	
*M. tilburgii*	2	1
*M. triplex*		1
*M. wolinsky*		1
MAC		3
TOTAL	130	408

### Summer

Of 1140 cultures processed in summer, only 1.6% were negative, 30.1% were overgrown, 50.2% were positive and 18.2% were positive with contaminants. Unfortunately, of the positive plates that were subcultured, a large percentage became contaminated and the mycobacterial yield was disappointing.

There was a wide variety of species identified using 16s rRNA sequencing (Table 3). Those isolates identified as *M. abscessus/M. chelonae* underwent subsequent *hsp*65 and *rpo*B gene fragment sequencing for further differentiation. Exhaustive speciation was not performed as only potentially pathogenic mycobacteria were of interest.

Overall there were more species identified in winter. All of the *M. intracellulare*, MAC, *M. lentiflavum, M. simiae*, *M. chelonae* isolates were found in winter, along with the majority of other pathogenic species such as *M. abscessus, M. kansasii, and M. mucogenicum. M. poriforae* and *M. fluoranthenivorans* were predominantly found in summer samples and *M. fortuitum* and *M. mucogenicum* were found equally in winter and summer.

### Decontamination

Decontamination made a statistically significant difference to culture results for all media used (p < 0.0001 for all). Overall decontamination did decrease the overgrowth and contamination of positive plates, and increased the yield from positive plates (Table 4).

**Table 4:** Effect of decontamination on culture results for different media

**Media**	**Decontamination**	**Culture result n (%)**				**Significance**
		**Negative**	**Positive**	**Positive + contaminants**	**Overgrown**	
MGIT Winter	No	43 (21.9)	35 (17.9)	115 (58.7)	3 (1.5)	p < 0.0001*
	Yes	99 (50.8)	49 (25.1)	46 (23.6)	1 (0.5)	
MGIT + PANTA Winter	No	4 (0.7)	48 (24.7)	82 (42.3)	3 (1.5)	p < 0.0001*
	Yes	14 (2.5)	64 (32.8)	19 (9.7)	1 (0.5)	
7H11 Summer	No	4 (0.7)	234 (41.1)	145 (25.4)	187 (32.8)	p < 0.0001
	Yes	14 (2.5)	338 (59.3)	62 (10.9)	156 (27.4)	
7H11 Winter	No	46 (7.9)	313 (53.5)	115 (19.7)	111 (19)	
	Yes	161 (27.5)	335 (57.3)	20 (3.4)	69 (11.8)	

The use of the MGIT tubes in winter increased the yield for certain species of mycobacteria. There were 13 isolates of *M. abscessus* – 10 of these were only grown using liquid media (7 MGIT + PANTA, 3MGIT). However the three isolates that grew on solid media were from sites that were not picked up by liquid media. *M. lentiflavum* was only identified in winter samples. Eight sites grew *M. lentiflavum* from MGIT only (1 MGIT, 7 MGIT + PANTA). It was grown on solid media from 6 sites – 4 of these sites also had positive MGITs (2) and MGIT + PANTA (3). For the majority of sites from which *M. gordonae* was identified, it was detected using M7H11. However, from ten sites it was only grown from MGIT tubes (4 MGIT + PANTA). Twenty-four sites grew *M. kansasii* only from MGIT (11 MGIT + PANTA only). Several of the pathogenic species shown in Table 5, were not grown in liquid media and only grew on solid media (eg *M. fortuitum, M. intracellulare*, *M. avium*).

**Table 5:** Number of NTM isolates cultured using different media

	**Winter media**			**Summer media**
**Species**	**MGIT**	**MGIT + PANTA**	**7H11**	**7H11**
*M. abscessus*	3	7	3	
*M. abscessus/chelonae*	1		2	
*M. bolletii/M. massiliense*		1		
*M. fortuitum complex*			14	13
*M. gordonae*	14	12	94	24
*M. kansasii*	34	45	52	5
*M. mucogenicum*	6	4	32	31
*M. terrae*				2
*M. intracellulare*			2	1
*M. lentiflavum*	3	10	6	
*MAC*			3	
*M. flavescens*			1	2
*M. interjectum*		1	6	1
*M. simiae*			2	
*M. szulgai*	1		9	

MGIT: Mycobacterial Growth Indicator Tube; PANTA: polymixin, azlocillin, naladixic acid, trimethroprim, amphotericin B.

## Discussion

This is the first study to document the presence of potentially pathogenic NTM in an Australian drinking water distribution system (DS). The incidence of disease is increasing [2] and water as a potential source of infection needs to be addressed. NTM have been reported in potable water studies from other countries. Mycobacteria were isolated from 38% (16/42) of drinking water DS in the USA [7], from 21.3% (42/197) in Greece [20] and 72% (104/144) in Paris [8]. Mycobacteria were found in Finnish DS samples – from 35%, up to 80% at sites more distal in the network [21]. Mycobacterial numbers reported are similar in DS that used groundwater compared to surface water [7]. In our study we identified NTM in samples from 82.1% of sites tested in winter and 40.2% sites in summer.

Kubalek demonstrated seasonal variations in the occurrence of environmental mycobacteria in potable water in the Czech Republic between 1984 and 1989 [22]. Forty two percent of samples were positive for mycobacteria, with significantly more positive in Spring than Autumn. We have similarly shown differences in seasonal isolation of NTM, and differences in the species isolated between seasons.

Factors associated with the isolation of pathogenic NTM included distance of sampling points from the main treatment plant, diameter of the pipes at point of sampling, and certain pipe materials. Pelletier found that free chlorine concentrations gradually decrease as water travels down the distribution system [23]. From previous studies [21,24] one would expect that mycobacterial growth would be greater the further from disinfection. Du Moulin found that communities in Massachusetts were more likely to have patients with MAC isolates if they lived further away from water treatment plants, and if they lived in more densely populated areas [25]. This can be explained by more complex water distribution systems in urban areas, with increased numbers of smaller diameter pipes, coupled with greater transit time of water in the system allowing for degradation of disinfection products.

By their hydrophobic nature, mycobacteria have the ability to form biofilms in pipes of distribution networks, contributing to their proliferation and survival [1]. Smaller pipes have a greater surface area of biofilms to contribute NTM into the water, and are usually present at more distal points of the system where complex bends occur. During pressure transients at point of turbulence such as the bends in pipes, release of biofilms occurs (sloughing). Falkinham [24] demonstrated significantly higher mycobacterial numbers in distribution samples (average 25000 fold) than those collected immediately downstream from treatment plants, indicating that mycobacteria actively grow within the distribution system. Whilst we didn’t find that smaller diameter pipes were more likely to yield NTM, pathogenic species more certainly more likely to come from sites with smaller diameter pipes.

Some pipe materials have been shown to contribute to biofilm formation particularly Iron pipes (compared to chlorinated PVC) [26]. However the survival of mycobacteria in DS is dependent upon a complex interaction between pipe surface, nutrient levels and disinfectants. In one study [27], when biofilms were grown on non-corroded surfaces (copper or PVC) free chlorine was more effective for controlling HPC and *M. avium*, but monochloramine controlled bacterial levels better on corroded iron pipe surfaces. *M. avium* biofilm levels were higher on iron and galvanized pipe surfaces than on copper or cPVC surfaces. In this study we were unable to assess the relative contribution of disinfectant concentrations, and nutrient levels, however there did seem to be some pipe surfaces (such as asbestos cement or modified PVC) associated with a greater yield of pathogenic mycobacteria at point of sampling. These results were consistent for both summer and winter, when chlorine concentrations may have been different (due to heat inactivation).

There was a wide variety of species isolated from water, many of which have been documented to cause disease in QLD patients [2]. *M. intracellulare* is the main pathogen associated with pulmonary disease in many parts of the world (including Australia and the United States) [28]. In our study, the isolation of *M. intracellulare* from water distribution samples was disappointing and similar to previous investigators. This has been attributed to the difficulties associated with culturing this organism from environmental samples as high concentrations have been found in biofilm samples from water meters or pipes [24]. However as disease associated serotypes of *M. intracellulare* have been found in soil and house dust, [29,30] and rainwater tanks, [31] the environmental niche for *M. intracellulare* may not necessarily be potable water, rather soil and dust contaminates water supplies through breaches in distribution systems (e.g. cracked underground pipes).

It has long been recognised that *M. kansasii* can be found in potable water [4,32,33]. Disease due to this organism is not common in Queensland (approximately 20 cases of significant pulmonary disease per year), yet this species was readily isolated from potable water.

*M. abscessus* has become an increasing clinical problem in the last 5–10 years, and its presence in potable water has not previously been emphasized. In the majority of published studies looking for NTM in water, no *M. abscessus* was documented. There have been taxonomical changes, which led to *M. abscessus* being recognised as independent from *M. chelonae*, so older studies reporting *M*. *chelonae* may have included *M. abscessus*. But in studies done since 2000 *M. abscessus* has been rarely reported [24,33-35]. The inclusion of liquid media in our study may have increased the yield for *M. abscessus*.

The universal problem with studies of environmental samples has been the difficulty in culturing these slow growing organisms in the presence of fungal and other bacterial contaminants [1,36]. Direct detection using PCR probes or a metagenomic approach is appealing however positive results may indicate the presence of mycobacterial DNA, but not necessarily viable organisms. This is especially relevant in the presence of disinfection, such as with potable water.

A major study examining showerheads in the USA using such an approach [37], did find *M. avium* and *M. gordonae* in multiple samples. *M. abscessus* was not reported.

## Conclusion

We have documented pathogenic NTM in the municipal drinking water distribution system of a major Australian city. Distance of sampling sites from treatment plants, narrower diameter pipes (predominantly distribution point sites) and sites with asbestos cement or modified PVC pipes were more likely to harbor pathogenic NTM. It is predicted that the interaction between humans and mycobacteria will increase, resulting in more cases of disease. Factors driving this increase include disinfection of drinking water with chlorine, selecting mycobacteria by reducing competition and the increasing percentage of our population with predisposing conditions, especially age and immunosuppression. Public and environmental health efforts must therefore focus on actions that will specifically remove mycobacteria from habitats where susceptible humans are exposed. Based on our findings, additional point chlorination, maintenance of more constant pressure gradients in the system, and the utilisation of particular pipe materials should be considered.

## Authors’ contributions

RT designed the study, coordinated the collection of samples, participated in the processing of water samples, collated and analysed the data, and wrote the manuscript. RC coordinated, received and processed the water samples (including subculturing and sequencing), collated the results and reviewed the manuscript. CT processed water samples, performed sequencing and collated results.CC contributed to the study design, provided institutional support and reviewed the manuscript. FH intellectually contributed to the study design and methodology and the writing of the manuscript. MH intellectually contributed to the study design and methodology, liaised with Brisbane Water, and contributed to the writing of the manuscript. All authors read and approved the final manuscript.

## Supplementary Material

Additional file 1: Table S1 Characteristics of the Brisbane Water distribution network. (From National Performance Report 2007–2008: urban water utilities. Downloaded 9/1/2012 from http://www.nwc.gov.au/publications Australian Government National Water Commission (page last updated 25/11/11). Click here for file

Additional file 2: Figure S1 Culture results according to pipe material at sampling site (complements Figure 2). **Table S2.** Site factors (Pipe diameter, mains age, elevation and distance from treatment plants) associated with culture result. Click here for file

Additional file 3 Species of NTM isolated from different sample types. Click here for file
