# Supplementary material for: Factors associated with the isolation of Nontuberculous mycobacteria (NTM) from a large municipal water system in Brisbane, Australia
Source: BMC Microbiol. 2013 Apr 22;13:89. doi: 10.1186/1471-2180-13-89 (PMC3651865; doi:10.1186/1471-2180-13-89)
Supplement: Additional file 1: Table S1 — Characteristics of the Brisbane Water distribution network. (From National Performance Report 2007–2008: urban water utilities. Downloaded 9/1/2012 from http://www.nwc.gov.au/publications Australian Government National Water Commission (page last updated 25/11/11). [file 1471-2180-13-89-S1.docx]

**Table S1**. Characteristics of the Brisbane Water distribution network. (From National Performance Report 2007-2008: urban water utilities. Downloaded 9/1/2012 from [www.nwc.gov.au/publications](http://www.nwc.gov.au/publications) Australian Government National Water Commission (page last updated 25/11/11)

|  | 2006-7 | 2007-8 |
| --- | --- | --- |
| Volume of water source from surface water (ML) | 11632 | 387 |
| Volume of water sourced from groundwater (ML) | 10 | 2268 |
| Volume of water sourced from recycling (ML) | 6155 | 5931 |
| Volume of water received from bulk supplier (ML)^[[1]](#footnote-1)^ | 140 049 | 129 226 |
| **Total sourced water** | **168846** | **137 812** |
| Total urban water supplied (ML) | 118 632 | 101 210 |
| Average annual residential water supplied (kL/property) | 153 | 128 |
| Number of water treatment plants | 4 | 4 |
| Length of water mains (km) | 6340 | 6369 |
| Properties served per km of water main | 69 | 69 |
| Population receiving water supply services (000s) | 1 006 | 1 021 |
| Total connected properties – water supply (000s) | 435 | 441 |
| **Water Quality Compliance** |  |  |
| Water Quality Guidelines | ADWG 2004 | ADWG 2004 |
| No. of zones where microbiological compliance achieved | 3 of 3 | 3 of 3 |
| % of population where microbiological compliance achieved | 100% | 100% |
| No. of zones chemical compliance achieved | 3 of 3 | 3 of 3 |

1. Over 75% of the water used in South East Queensland (SEQ) comes from three dams operated by SEQwater (Wivenhoe, Somerset and North Pine) [↑](#footnote-ref-1)
