# Supplementary material for: Factors associated with the isolation of Nontuberculous mycobacteria (NTM) from a large municipal water system in Brisbane, Australia
Source: BMC Microbiol. 2013 Apr 22;13:89. doi: 10.1186/1471-2180-13-89 (PMC3651865; doi:10.1186/1471-2180-13-89)
Supplement: Additional file 2: Figure S1 — Culture results according to pipe material at sampling site (complements Figure 2). Table S2. Site factors (Pipe diameter, mains age, elevation and distance from treatment plants) associated with culture result. [file 1471-2180-13-89-S2.docx]

**Additional Material 2**

**Figure S1:**. Pipe materials of different types of samples.


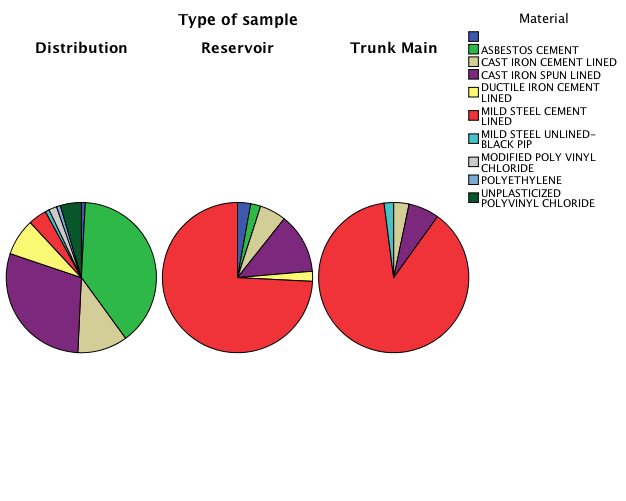


| **Table S2: Characteristics of different types of samples.** | | | | | | |
| --- | --- | --- | --- | --- | --- | --- |
| Type of sample | | N | Minimum | Maximum | Mean | Std. Deviation |
| **Distribution** | Diameter (mm) | 1850 | 80.00 | 1670.00 | 185.46 | 230.41 |
|  | Main age (years) | 1850 | 3 | 103 | 48.79 | 21.46 |
|  | Elevation (m AHD) | 1639 | 0.00 | 143.42 | 32.25 | 24.45 |
|  | Distance to nearest reservoir (km) | 1639 | 0.02 | 24.49 | 5.71 | 5.76 |
| **Reservoir** | Diameter (mm) | 725 | 100.00 | 1670.00 | 691.83 | 466.14 |
|  | Main age (years) | 725 | 16 | 88 | 44.86 | 14.92 |
|  | Elevation (m AHD) | 666 | 17.58 | 283.68 | 80.75 | 53.65 |
|  | Distance to nearest reservoir (km) | 666 | 0.01 | 21.81 | 0.66 | 3.34 |
| **Trunk Main** | Diameter (mm) | 484 | 100.00 | 1670.00 | 984.92 | 392.93 |
|  | Main age (years) | 484 | 34 | 119 | 50.80 | 15.67 |
|  | Elevation (m AHD) | 382 | 0.00 | 94.27 | 27.42 | 21.42 |
|  | Distance to nearest reservoir (km) | 382 | 0.08 | 20.46 | 9.72 | 7.08 |
|  |  |  |  |  |  |  |
