# Supplementary material for: Factors associated with the isolation of Nontuberculous mycobacteria (NTM) from a large municipal water system in Brisbane, Australia
Source: BMC Microbiol. 2013 Apr 22;13:89. doi: 10.1186/1471-2180-13-89 (PMC3651865; doi:10.1186/1471-2180-13-89)
Supplement: Additional file 3 — Species of NTM isolated from different sample types. [file 1471-2180-13-89-S3.docx]

| **Species** | **Trunk main**  **n= 484** | **Reservoir**  **n= 725** | **Distribution**  **n=1850** |
| --- | --- | --- | --- |
| *M. abscessus* | 1 (1.4) | 1 (0.8) | 12 (3.6) |
| *M. angelicum/szulgai* |  |  | 1 (0.3) |
| *M. arupense* | 3 (4.1) | 2 (1.5) | 4 (1.2) |
| *M. austroafricanum* |  |  | 1 (0.3) |
| *M. bolletii/M. massiliense* |  |  | 1 (0.3) |
| *M. chelonae* | 1 (1.4) |  | 1 (0.3) |
| *M. cookii* |  |  | 2 (0.6) |
| *M. cosmeticum* | 1 (1.4) |  | 1 (0.3) |
| *M. diernhoferi* | 1 (1.4) | 3 (2.3) |  |
| *M. farcinogenes* |  | 1 (0.8) | 2(0.6) |
| *M. flavescens* |  | 1 (0.8) | 2 (0.6) |
| *M. fluoranthenivorans* | 2 (2.7) | 4 (3.1) | 9 (2.7) |
| *M. fortuitum complex* | 1 (1.4) | 4 (3.1) | 22 (6.6) |
| *M. gadium* | 1 (1.4) | 1 (0.8) | 3 (0.9) |
| *M. gilvum* |  |  | 1 (0.3) |
| *M. gordonae* | 25 (33.8) | 51 (38.9) | 68 (20.8) |
| *M. interjectum* | 2 (2.7) |  | 6 (1.8) |
| *M. intracellulare* |  | 1 (0.8) | 1 (0.3) |
| *M. kansasii* | 19 (25.7) | 30 (22.9) | 89 (26.8) |
| *M. lentiflavum* | 1 (1.4) | 3 (2.3) | 15 (4.5) |
| *M. mageritense* | 1 (1.4) |  | 4 (1.2) |
| *M. moriokaense* | 1 (1.4) |  |  |
| *M. mucogenicum* | 9 (12.2) | 15 (11.5) | 49 (14.8) |
| *M. poriforae* |  | 8 (6.1) | 15 (14.5) |
| *M. rhodesiae* |  | 1 (0.8) |  |
| *M. sengalense* | 1 (1.4) |  | 1 (0.3) |
| *M. simiae* | 1 (1.4) |  | 1 (0.3) |
| *M. species NFI* | 1 (1.4) | 2 (1.5) | 6 (1.8) |
| *M. szulgai* | 1 (1.4) | 1 (0.8) | 8 (2.4) |
| *M. terrae* |  | 1 (0.8) | 1 (0.3) |
| *M. tilburgii* | 1 (1.4) | 1 (0.8) | 1 (0.3) |
| *M. triplex* |  |  | 1 (0.3) |
| *M. wolinsky* |  |  | 1 (0.3) |
| MAC |  |  | 3 (0.9) |
| Total | 74 | 129 | 332 |

**Additional file 3:** Species of NTM isolated from different sample types.
